# Supplementary material for: Changes in rapid plasma reagin titers in patients with syphilis before and after treatment: A retrospective cohort study in an HIV/AIDS referral hospital in Tokyo
Source: PLoS One. 2023 Sep 28;18(9):e0292044. doi: 10.1371/journal.pone.0292044 (PMC10538775; doi:10.1371/journal.pone.0292044)
Supplement: Attachment — Submitted filename: Response_230703.doc [file pone.0292044.s003.doc]

We replied to each comment as follows.

**Reviewer 1**

1. Line 64-66: Please, try to update the bibliography as, for instance, reference [2] goes back to 1968. There are several most recent manuscripts that can be cited here [Tiecco G, Pathogens. 2021 or Ramchandani MS, Infect Dis Clin North Am. 2023].

Response: Thank you for your advice. We have added the reference you suggested (Line 66).

2. Line 154-160: Please, add percentage in the main text in order to make data more easily comparable.

Response: Thank you for your advice. We have added the percentage of included patients (Line 155-158).

3. Line 232-236: Please, expand the discussion by including also other clinical conditions that might influence the serological response, and, if it is possible, compare your data to the available literature. Post-treatment RPR might be different in case of first infection treatment or syphilis reinfection management [Marchese V, J Clin Med. 2022]. Moreover, several atypical conditions might exist especially in PLWH such as serological-non-responder or serofast status [Seña AC, BMC Infect Dis. 2015 or Ghanem KG, Sex Transm Dis. 2021].

Response: Thank you for your valuable comments. We have added an explanation addressing the potential impact of prior syphilis infection on post-treatment RPR change and the potential influence of serofast and serological non-responder, which are commonly observed in HIV patients. We have also added the references you suggested (Lines 242-246).

Sincerely,

Kazuaki Fukushima, MD.
