# Supplementary material for: Changes in rapid plasma reagin titers in patients with syphilis before and after treatment: A retrospective cohort study in an HIV/AIDS referral hospital in Tokyo
Source: PLoS One. 2023 Sep 28;18(9):e0292044. doi: 10.1371/journal.pone.0292044 (PMC10538775; doi:10.1371/journal.pone.0292044)
Supplement: Attachment — Submitted filename: Response to reviewers_230801.doc [file pone.0292044.s004.doc]

In the second round of review in June (PONE-D-22-35264R1), Reviewer 1 provided the following comment regarding the discussion section. Based on this comment, we have revised the discussion section in response to the previous revision, and we apologize for any insufficiencies in our previous revisions.

Consequently, we have carefully addressed these comments and made revisions to the discussion section accordingly.

**Reviewer 1**

Comment 3. Line 232-236: Please, expand the discussion by including also other clinical conditions that might influence the serological response, and, if it is possible, compare your data to the available literature. Post-treatment RPR might be different in case of first infection treatment or syphilis reinfection management [Marchese V, J Clin Med. 2022]. Moreover, several atypical conditions might exist especially in PLWH such as serological-non-responder or serofast status [Seña AC, BMC Infect Dis. 2015 or Ghanem KG, Sex Transm Dis. 2021].

Based on this comment, we revised the discussion section. In this latest revision, we have focused on the factors that influence the speed of RPR decrease after treatment and conducted a thorough comparison of our study with relevant references (Lines 234-255). Additionally, we have added a new paragraph about the complex effect of HIV on RPR dynamics based on provided references (Lines 256-265).

Thank you once again for your careful consideration of our work. We look forward to receiving further guidance from you regarding the next steps in the publication process.

The authors have no conflicting financial interests, all authors concur with the submission and have contributed to this work, and the material submitted has not been previously reported nor is under consideration for publication elsewhere.

Sincerely,

Kazuaki Fukushima, M.D.
